# Supplementary material for: Association between oral microbiome and seven types of cancers in East Asian population: a two-sample Mendelian randomization analysis
Source: Front Mol Biosci. 2023 Nov 21;10:1327893. doi: 10.3389/fmolb.2023.1327893 (PMC10702768; doi:10.3389/fmolb.2023.1327893)
Supplement: Supplementary file 1 [file DataSheet1.ZIP › Supplementary Materials/MR plots of saliva/saliva═╝/colorectal cancer/pheno.2110_to_colorectal cancer_forest.pdf]

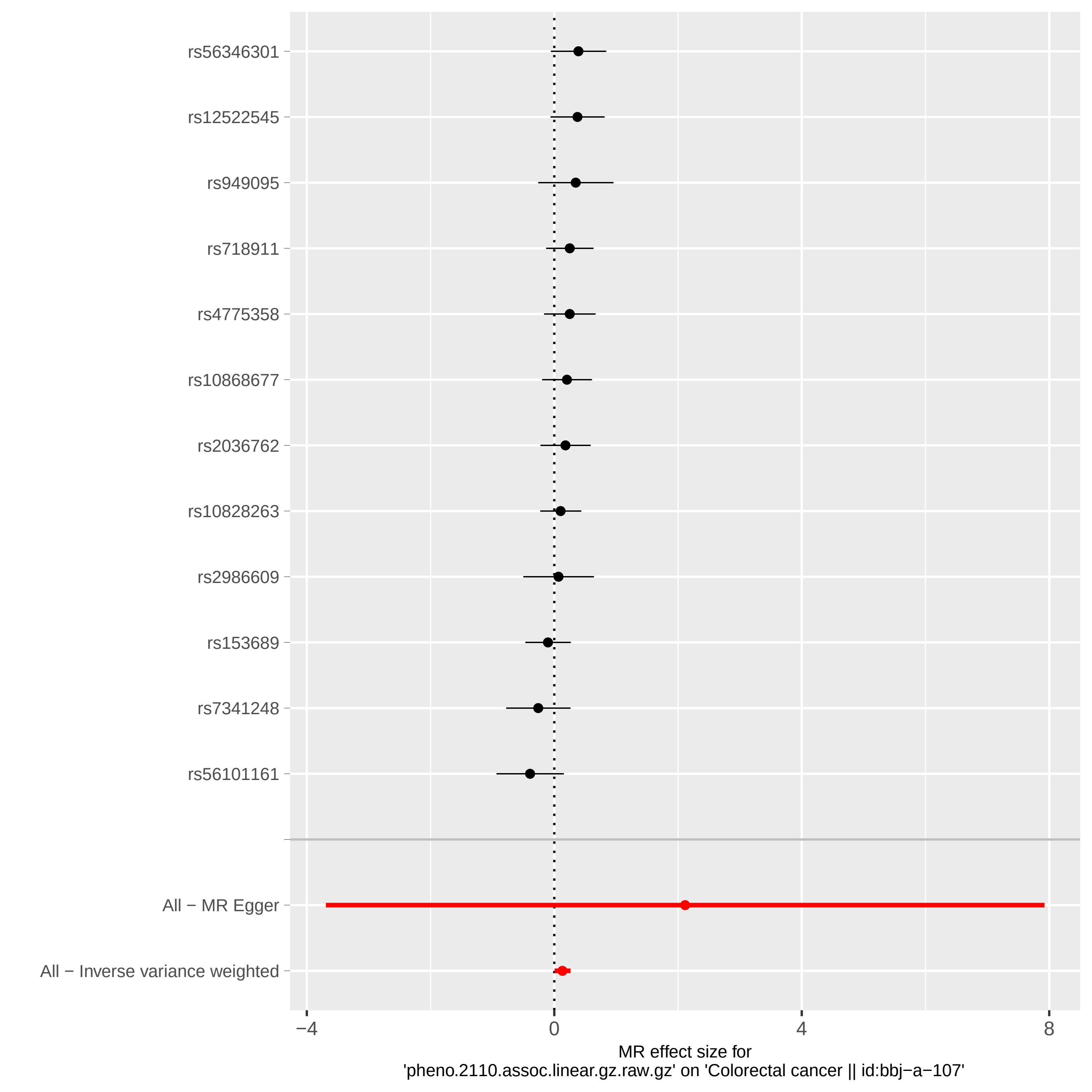
rs56346301
rs12522545
rs949095
rs718911
rs4775358
rs10868677
rs2036762
rs10828263
rs2986609
rs153689
rs7341248
rs56101161
All − MR Egger
All − Inverse variance weighted
−4
0
4
8
MR effect size for
'pheno.2110.assoc.linear.gz.raw.gz' on 'Colorectal cancer || id:bbj−a−107'
